# Manipulating Microtubules in Systemic Sclerosis

**DOI:** 10.1371/journal.pmed.0020403

**Published:** 2005-11-01

Systemic sclerosis (scleroderma or SSc) is the name for a group of progressive diseases, all of which involve the abnormal growth of connective tissue. They are chronic degenerative disorders in which there is widespread vascular deterioration and tissue loss. The recognition of SSc dates back many years. Indeed, the characteristic stretched thickening of the skin was probably first described by Hippocrates. A more definitive description of the condition was made by Carlo Curzio in 1753, who described a patient as having wood-like skin with “tight eyelids, difficulty in opening her mouth, coldness of her skin.”

The etiology of SSc is still not completely understood, but appears to be autoimmune. Downstream of the immune activation, the molecules that have been implicated are the profibrotic cytokines, such as transforming growth factor-beta (TGFβ), interleukin-4 (IL-4), platelet-derived growth factor (PDGF), and connective tissue growth factor, all of which can cause fibrosis. In addition to the profibrotic effects, TGFβ and PDGF may also contribute to vasculopathy. Furthermore, the vascular changes in SSc skin lesions are associated with anti-endothelial cell autoantibodies.

One problem in the research on SSc has been finding an appropriate animal model. One such model has now been developed: the transplantation of skin samples from patients with SSc into immunodeficient mice. Without the interference of an effective murine immune system, it is possible to test the effect of various interventions on the patients' skin samples. [Fig pmed-0020403-g001]

**Figure pmed-0020403-g001:**
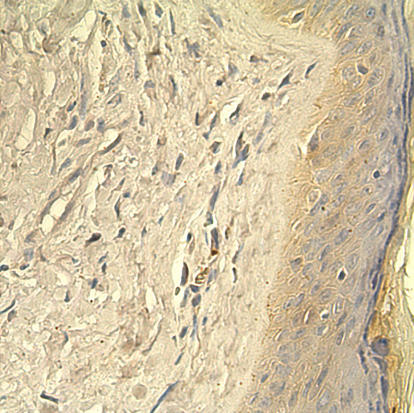
Suppression of Smad2 phosphorylation by paclitaxel in in SSc skin graft

In a paper in *PLoS Medicine*, Chunming Dong and colleagues used this model to investigate the effect of paclitaxel on SSc skin samples. Paclitaxel is an attractive drug because it stabilizes microtubules, which affect the propagation of TGFβ signaling. TGFβ is a multifunctional regulatory cytokine involved in a large number of cellular activities. It initiates its effects by binding to and activating specific cell-surface receptors. These activated TGFβ receptors stimulate a family of proteins (R-Smads), some of which act to stimulate further TGFβ signaling, and others of which act to inhibit it. The microtubules regulate the R-Smad access to and activation by TGFβ receptors.

Dong and colleagues were able to show that in this model of SSc, paclitaxel markedly suppressed the activation of two of the Smads, Smad2 and Smad3, and, hence, collagen deposition in SSc grafts. They also showed that the SSc grafts had increased neovessel formation relative to normal grafts, regardless of paclitaxel treatment, thus indicating that paclitaxel did not have a negative effect on angiogenesis. In fact, they found that the neovascular cells were likely derived from the mouse hosts.

Do these findings indicate that paclitaxel is a good drug for SSc? Certainly more work needs to be done before this can be said. A particular concern that paclitaxel can itself lead to fibrosis at high doses when used, for example, in the treatment of certain tumors (in contrast to low doses used here) needs to be assessed further. However, SSc is a difficult progressive disease, and results such as these should be considered seriously as avenues for future therapies.

